# High throughput estimates of *Wolbachia*, Zika and chikungunya infection in *Aedes aegypti* by near-infrared spectroscopy to improve arbovirus surveillance

**DOI:** 10.1038/s42003-020-01601-0

**Published:** 2021-01-15

**Authors:** Lilha M. B. Santos, Mathijs Mutsaers, Gabriela A. Garcia, Mariana R. David, Márcio G. Pavan, Martha T. Petersen, Jessica Corrêa-Antônio, Dinair Couto-Lima, Louis Maes, Floyd Dowell, Anton Lord, Maggy Sikulu-Lord, Rafael Maciel-de-Freitas

**Affiliations:** 1 Laboratório de Transmissores de Hematozoários, IOC, Fundação Oswaldo Cruz, Rio de Janeiro, RJ 21040-360 Brazil; 2 Laboratory for Microbiology, Parasitology and Hygiene (LMPH), University of Antwerp, 2000 Antwerp, Belgium; 3 Instituto Nacional de Ciência e Tecnologia em Entomologia Molecular (INCT-EM), Universidade Federal do Rio de Janeiro, Rio de Janeiro, RJ 21941-902 Brazil; 4 USDA-ARS, Center for Grain and Animal Health Research, Manhattan, KS 66502 USA; 5 The School of Public Health, The University of Queensland, Herston, QLD 4006 Australia; 6 QIMR Berghofer Medical Research Institute, Herston, QLD 4006 Australia

**Keywords:** Pathogens, Infectious-disease diagnostics

## Abstract

Deployment of *Wolbachia* to mitigate dengue (DENV), Zika (ZIKV) and chikungunya (CHIKV) transmission is ongoing in 12 countries. One way to assess the efficacy of *Wolbachia* releases is to determine invasion rates within the wild population of *Aedes aegypti* following their release. Herein we evaluated the accuracy, sensitivity and specificity of the Near Infrared Spectroscopy (NIRS) in estimating the time post death, ZIKV-, CHIKV-, and *Wolbachia*-infection in trapped dead female *Ae. aegypti* mosquitoes over a period of 7 days. Regardless of the infection type, time post-death of mosquitoes was accurately predicted into four categories (fresh, 1 day old, 2–4 days old and 5–7 days old). Overall accuracies of 93.2, 97 and 90.3% were observed when NIRS was used to detect ZIKV, CHIKV and *Wolbachia* in dead *Ae. aegypti* female mosquitoes indicating NIRS could be potentially applied as a rapid and cost-effective arbovirus surveillance tool. However, field data is required to demonstrate the full capacity of NIRS for detecting these infections under field conditions.

## Abstract (web summary)

Santos et al. demonstrate that the Near Infrared Spectroscopy (NIRS) can accurately estimate the death time of trapped female *Aedes aegypti* and vector infection with Zika virus, Chikungunya virus, or Wolbachia in a 7-day trapping period. This study suggests that NIRS may provide an accurate and inexpensive tool that improves arbovirus surveillance systems.

## Introduction

Almost half of the global human population currently live in areas under risk of arbovirus transmission^1^. The incidence of mosquito-borne arboviruses, such as dengue (DENV), Zika (ZIKV), and chikungunya (CHIKV) has been on the rise globally in the past decade due to increased geographical spread of the primary vector *Aedes aegypti* and the secondary vector *Aedes albopictus* to places where they were originally absent^2^. Arboviruses outbreaks are linked to favorable climatic conditions, entomological, epidemiological, and immunological factors. The absence of an effective vaccine and a lack of effective and timely vector surveillance system are major contributors of rapid arbovirus spread^3^, which ultimately pose a high economic burden, including severe disability-adjusted life year to affected populations^4–6^.

Monitoring the spatial and temporal dynamics of vector populations through larval surveys remains a routine activity in many countries^7^. Although sampling adult mosquitoes provides more reliable estimates of *Aedes* population size and allows stratifying areas according to the risk of outbreaks^8,9^, estimation of transmission risk rely mostly on monitoring vector density as opposed to virus density in wild mosquitoes. Determining arbovirus infection in wild caught *Aedes* mosquitoes is rarely done by most vector control programs. This is because the positivity rate of captured mosquitoes is in most cases <1% and therefore setting up a large-scale routine surveillance system to detect such a natural infection in trapped mosquitoes is costly, time consuming, and unfeasible in several endemic countries^10–14^. Nonetheless, predicting when and where the next epidemic will strike is of utmost importance to facilitate vector control intensification in areas where risk is highest. Hence, surveillance methods should be capable of providing rapid detection of arboviruses required to initiate an effective response against arboviral threats^15^. In such a scenario, a rapid, accurate, and cost-effective tool will facilitate early arbovirus detection in trapped mosquitoes to trigger early warning systems, which will in turn initiate a timely intervention^16^.

One of the most promising ongoing intervention against arboviruses is releasing *A. aegypti* mosquitoes transinfected with the maternally inherited endosymbiotic bacterium *Wolbachia pipientis* in the field. This bacterium works by manipulating host reproduction through cytoplasmic incompatibility resulting in nonviable offspring, when uninfected females mate with *Wolbachia*-infected males^17–19^. *Wolbachia*-infected *A. aegypti* can block transmission of several arboviruses, including DENV^18,20,21^, ZIKV^22,23^, CHIKV, Mayaro, and yellow fever virus^24,25^. The ongoing use of *Wolbachia* in >14 countries is based on replacing the highly susceptible native *A. aegypti* population with the less susceptible *Wolbachia*-infected *A. aegypti* population to ameliorate arboviral transmission^26–28^. Population replacement is achieved after releasing hundreds of thousands of *Wolbachia*-infected *A. aegypti* mosquitoes. The prevalence of *Wolbachia* in the field is assessed through screening trapped mosquitoes. Such information is intended to guide subsequent releases. For example, whether to increase or reduce the number of insects in future releases or even stop the release if a desired frequency has been achieved. Traditionally, adult mosquito trapping is carried out with BG-Sentinel traps, but ovitraps are sometimes used concomitantly, usually on a weekly basis^26,27,29^. Collected samples (adult mosquitoes or eggs allowed to hatch in an entomological lab) are often screened with quantitative polymerase chain reaction (qPCR) technique to check for the presence of *Wolbachia*, but arboviruses presence in *Wolbachia*-infected and wild *A. aegypti* is rarely assessed^27^. Traps are inspected once a week in most cases, and thus the majority of trapped mosquitoes have died and dried out for period of time varying between 1 and 7 days^27^. This presents a big challenge in the detection of arboviruses and bacteria using conventional molecular techniques, such as PCR^30^.

The near-infrared spectroscopy (NIRS) technique has been demonstrated in a number of entomological studies as rapid, cost-effective, and high-throughput tool for characterizing biological samples based on spectral signatures. The spectral signatures mirror the amount of light reflected back following its absorption by C–H, O–H, S–H, and N–H functional groups present in those samples at specific frequencies. The type and concentration of these chemicals are unique to biological samples, and hence each unique sample can produce a specific diagnostic spectrum that can be analyzed by chemometrics or machine learning techniques to identify them. For this purpose, NIRS is: (a) non invasive, therefore the material can be used multiple times, (b) low cost, as it does not require reagents to operate and (c) rapid, as a spectrum can be collected in just 3 s, therefore allowing hundreds of samples to be analyzed daily. NIRS has been demonstrated for age prediction, species identity and for the detection of the presence of *Plasmodium falciparum* of the major African malaria vectors, *Anopheles gambiae* and *Anopheles arabiensis* under laboratory, semi-field, and field settings^31–34^. NIRS has also been used to predict the age of *A. aegypti*^35^, *A. albopictus*^36^, and to detect ZIKV in *A. aegypti*^37^, and *Wolbachia* in *A. aegypti*^38^ and in fruit flies^39^.

As a potential next-generation surveillance tool, NIRS could provide a reliable and accurate alternative to age grading, and diagnosis of arboviruses and *Wolbachia* in *A. aegypti*. Furthermore, NIRS could potentially be used to evaluate the spatiotemporal shifts in arbovirus transmission following *Wolbachia* deployment to allow health managers to rapidly and cost effectively assess the impacts of *Wolbachia* in reducing disease transmission and outbreaks. So far, the accuracy of NIRS for age grading and pathogen detection has mainly been demonstrated on fresh or preserved samples^40,41^. Therefore, the value of NIRS for detecting pathogens in mosquitoes that have been dead in a trap for a period of 7 days is yet to be demonstrated. To develop accurate NIRS models for predicting the presence of viruses and *Wolbachia* in a dead mosquito, ideally the approximate time of death should be known. The main objective of this study was to determine whether NIRS can estimate the approximate death time, and use this information to predict the presence of ZIKV, CHIKV, and *Wolbachia* in mosquitoes left in a BG-Sentinel trap for a period of 7 days post death.

## Results

### Confirmation of ZIKV, CHIKV, and *Wolbachia* in mosquitoes

Out of the 157, 59, and 163 mosquitoes screened for ZIKV, CHIKV, and *Wolbachia*, infection was confirmed in 82 (52.3%), 36 (61%), and 163 (100%) of them, respectively. At 7 days post death, 53 (54.6%) of ZIKV-infected mosquitoes were still positive for CHIKV.

### NIRS prediction of days post death

**Fig. 1:**
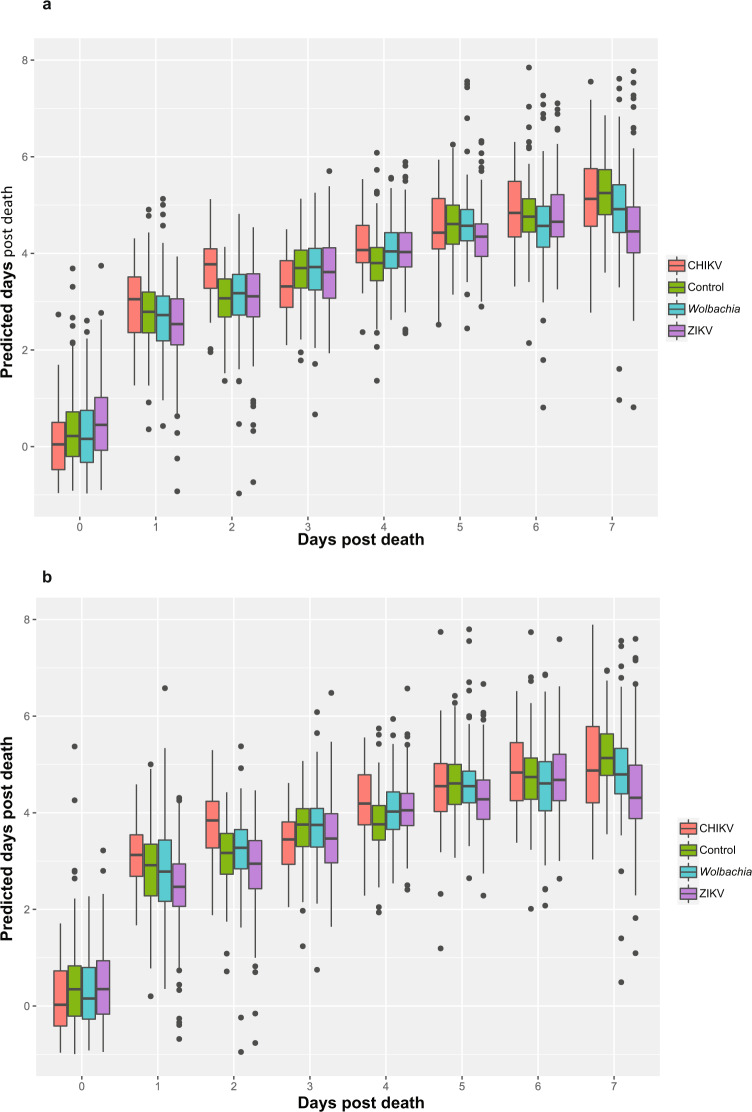
Death prediction model by days post death and infection status. Results from training data (**a**) and results from testing data (**b**). Monte Carlo simulations were performed using a 75%/25% training/testing split to validate the model. Infection status including CHIKV (red), ZIKV (purple), *Wolbachia* (blue), and uninfected controls (green) is presented in both panels. Box and whisker plots follow the standard convention where the box represents the range between quartiles 1 and 3, while the whiskers represent maximum and minimum excluding outliers. Outliers are defined as either Q1 − 1.5 × IQR or Q3 + 1.5 × IQR.

**Fig. 2:**
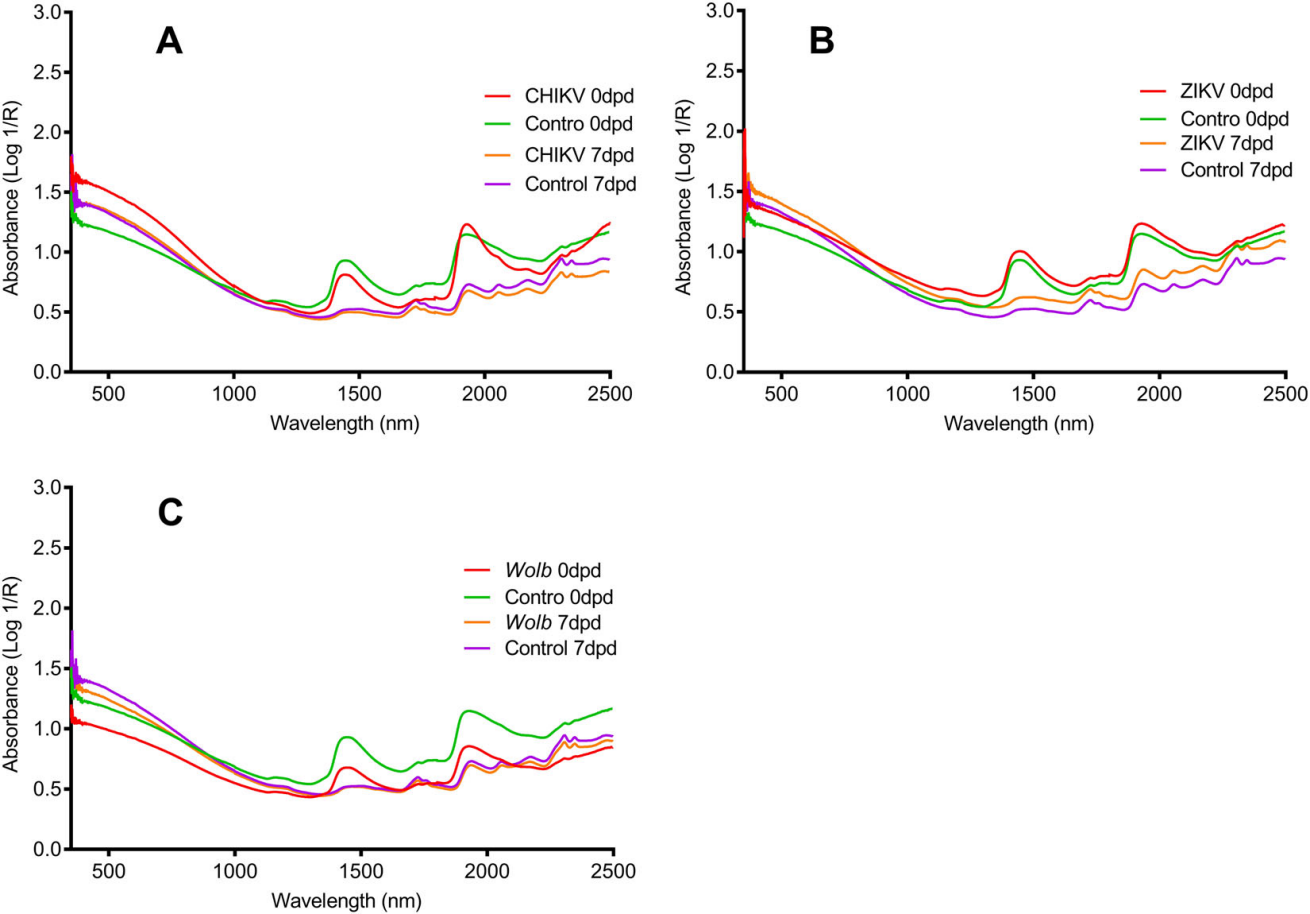
Changes in raw spectra of *A. aegypti* mosquitoes. CHIKV-infected and uninfected mosquitoes at day 0 and day 7 post death (**a**), ZIKV-infected and uninfected mosquitoes at day 0 and day 7 post death (**b**), and *Wolbachia*-infected and uninfected mosquitoes at day 0 and day 7 post death (**c**).

The training and testing model consisted of ZIKV (*n* = 157), CHIKV (*n* = 59), *Wolbachia* (*n* = 163), and uninfected mosquitoes (*n* = 129). Monte Carlo simulations were performed using a 75%/25% training/testing split to validate the model. The mean absolute error (MAE; standard deviation) for prediction of days post death in the training group was 1.16 ± 0.88, 1.24 ± 0.93, 1.18 ± 0.93, and 1.15 ± 0.82 for ZIKV, CHIKV, *Wolbachia*, and control mosquitoes, respectively (Fig. 1a). MAE (standard deviation) for days post death in the testing group were 1.25 ± 0.98, 1.27 ± 0.95, 1.34 ± 1.71, and 1.15 ± 0.84 for ZIKV, CHIKV, *Wolbachia*, and control mosquitoes, respectively (Fig. 1b). When considering the accuracy of NIRS for predicting days post death of all mosquitoes regardless of their infection type, they all grouped into four categories (fresh, 1 day old, 2–4 days old and 5–7 days old). Predicted scores for each group for all infections followed a similar trend, indicating that infection type does not affect NIRS days post death prediction accuracy (Fig. 2).

### NIRS prediction of CHIKV infection

**Fig. 3:**
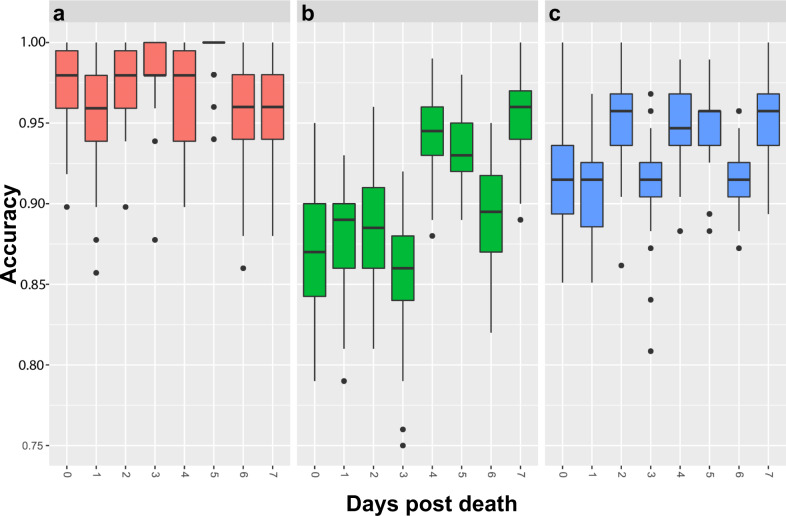
Prediction accuracy (test set) for detecting infection in *A. aegypti* mosquitoes. Mosquitoes infected with CHIKV (**a**), *Wolbachia* (**b**), and ZIKV (**c**) vs. uninfected mosquitoes. Box and whisker plots follow the standard convention where the box represents the range between quartiles 1 and 3, while the whiskers represent maximum and minimum excluding outliers. Outliers are defined as either Q1 − 1.5*IQR or Q3 + 1.5*IQR.

Models for predicting CHIKV were the most accurate, ranging from 95.1% to 99.8% accuracy depending on days post death the mosquitoes were scanned (Fig. 3a and Table 1). The average accuracy for predicting the presence or absence of CHIKV in mosquitoes regardless of when they died was 97%. Mosquitoes that were scanned on day 1 and day 7 post death were least accurately predicted, whereas mosquitoes that were scanned 5–6 days post death were predicted with a slightly higher accuracy. However, this accuracy did not differ statistically from the accuracy of fresh mosquitoes for all groups (*P* = 0.261). The overall sensitivity of NIRS for predicting CHIKV in mosquitoes regardless of time of death of a mosquito was 95.8%. This sensitivity ranged from 91.6% to 99.2% depending on the time post death the mosquitoes were scanned, and it was similar to the sensitivity observed for fresh mosquitoes (*P* = 1 in all cases). Similarly, specificity ranged from 95.6% to 99.8% depending on time post death the mosquitoes were scanned, and it did not significantly differ from fresh mosquitoes for all groups (*P* = 0.269; Table 1).

**Table 1:** Accuracy (Acc), sensitivity (Sens), specificity (SPC), and their respective *P* values of NIRS diagnostic models for ZIKV, CHIKV, and *Wolbachia* for 0–7 days post death.

Infection Type	Zika						Chikungunya						Wolbachia					
Days post death	%Acc	*P* value	%Sens	*P* value	%SPC	*P* value	%Acc	*P* value	%Sens	*P* value	%SPC	*P* value	%Acc	*P* value	%Sens	*P* value	%SPC	*P* value
0	91.8 (2.8)	Ref.	87.7 (5.2)	Ref.	94.5 (3.1)	Ref.	97.3 (2.3)	Ref.	96.7 (4.6)	Ref.	97.7 (2.4)	Ref.	87.1 (3.8)	Ref.	77.7 (6.8)	Ref.	93.0 (2.7)	Ref.
1	90.7 (3.1)	0.233	83.3 (5.7)	1.000	95.7 (2.5)	0.155	95.1 (3.2)	0.364	91.6 (6.5)	1.000	96.9 (3.1)	0.647	88.2 (3.4)	0.802	80.0 (5.7)	1.000	93.0 (3.2)	1.000
2	95.1 (2.4)	0.119	90.4 (4.5)	0.559	98.1 (1.9)	0.044	97.3 (2.2)	0.270	95.9 (4.4)	1.000	98.0 (2.0)	0.269	88.3 (3.6)	0.797	78.7 (6.2)	0.871	94.6 (3.3)	1.000
3	91.3 (2.8)	0.233	86.2 (5.2)	0.930	94.6 (2.7)	0.521	98.5 (2.1)	0.364	99.1 (2.8)	1.000	98.3 (2.3)	0.647	85.8 (3.5)	0.811	72.0 (5.2)	1.000	96.9 (2.5)	1.000
4	94.7 (2.4)	0.184	90.1 (4.3)	0.610	97.2 (2.4)	0.294	96.8 (2.7)	0.364	95.5 (5.2)	1.000	97.5 (2.5)	0.647	94.5 (2.5)	0.659	90.4 (5.2)	0.031	96.9 (2.5)	0.256
5	94.7 (2.1)	0.030	92.0 (4.7)	0.106	96.5 (2.3)	0.040	99.6 (1.2)	0.261	99.2 (3.4)	1.000	99.8 (0.8)	1.000	93.4 (2.1)	0.776	88.2 (4.7)	0.340	96.4 (2.1)	1.000
6	91.8 (2.1)	0.077	86.2 (4.0)	0.299	95.5 (2.3)	0.042	99.6 (3.2)	0.448	96.1 (4.7)	1.000	95.6 (3.8)	0.480	89.3 (3.3)	0.755	79.9 (5.6)	0.186	95.6 (2.8)	0.928
7	95.0 (2.6)	0.030	91.6 (4.5)	0.106	97.1 (2.5)	0.040	95.6 (3.0)	0.448	92.1 (6.7)	1.000	97.4 (2.9)	0.480	95.3 (2.5)	0.722	88.9 (5.2)	0.154	99.2 (1.5)	0.226
Overall	93.2 (3.1)	NA	88.5 (5.6)	NA	96.1 (2.8)	NA	97.0 (2.9)	NA	95.8 (5.5)	NA	97.7 (2.8)	NA	90.3 (4.6)	NA	81.9 (8.3)	NA	95.7 (3.3)	NA

Analysis was run 50 times using Monte Carlo sampling. Numbers represent the mean (standard deviation) of each test. Two sample *t* tests were performed between the accuracy of correctly predicting infectivity status using fresh (dead) mosquitoes vs. every other day sequentially. *P* values are not corrected for multiple comparisons.

### NIRS prediction of *Wolbachia* infection

The prediction accuracy of NIRS for detecting *Wolbachia* in *A. aegypti* ranged between 85.8% and 95.3% for the testing cohort (Fig. 3b and Table 1) with the average accuracy of 90.3%. The accuracy was not statistically different for all tested groups relative to fresh mosquitoes (*P* = 0.722). Mosquitoes were more accurately predicted (accuracy 95.3%) when scanned 7 days post death. Similarly, sensitivity and specificity values were higher when mosquitoes were scanned 7 days post death. Although not significantly different from fresh mosquitoes, sensitivity and specificity for *Wolbachia* detection increased when mosquitoes were scanned 4 days post death, and the overall sensitivity and specificity regardless of when mosquitoes were scanned was 81.9% and 95.7%, respectively (Fig. 3c and Table 1).

### NIRS prediction of ZIKV infection

Regardless of time post death, the average prediction accuracy of NIRS for detecting ZIKV was 93.2% for all mosquitoes scanned for the testing set. This accuracy ranged between 90.7% and 95.1% depending on the time post death mosquitoes were scanned (Fig. 3c and Table 1). The accuracy of predicting mosquitoes 5 and 7 days post death was higher and significantly different from the accuracy obtained when mosquitoes were scanned fresh (*P* = 0.03). The highest paired sensitivity and specificity were 92.0% and 96.5%, respectively, and they were achieved when mosquitoes were scanned 5 days post death. Overall sensitivity and specificity of 88.5% and 96.1% were achieved regardless of when the mosquitoes were scanned (Table 1).

### Prediction of infection in fresh vs. mosquitoes that were scanned 7 days post death

**Fig. 4:**
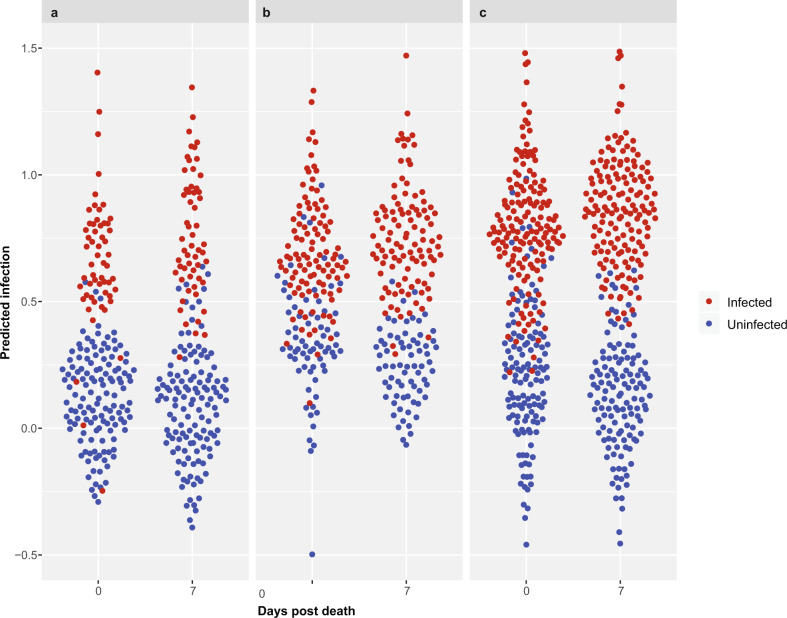
Prediction scores of uninfected and uninfected mosquitoes for 0 days post death and 7 days post death. Infection scores are shown for CHIKV (**a**), *Wolbachia* (**b**), and ZIKV (**c**), and each dot represents one individual. The *Y*-axis represents the prediction scores on a continuous scale prior to categorizing into >0.5 (predicted infected) and ≤0.5 (predicted uninfected).

When comparing overall accuracy, sensitivity, and specificity of ZIKV, CHIKV, and *Wolbachia*-infected mosquitoes scanned at 0 days post death (fresh) and those scanned 7 days post death, no difference was observed for CHIKV-infected mosquitoes. However, ZIKV and *Wolbachia*-infected mosquitoes were more accurately predicted 7 days post death than when they were scanned while fresh (Table 1 and Fig. 4).

## Discussion

An efficient arbovirus surveillance system monitors the early signs of viral circulation in human population, which would enable a timely response to prevent future disease outbreaks. The development of a rapid and cost-effective tool to identify the presence of arboviruses in mosquitoes is crucial for control of these arboviruses^16,42,43^, and can minimize disease outbreaks and assist in the timely reinforcement of vector control interventions^44–47^. Secondly, a tool that can detect infection in mosquitoes that have been left in a trap for an extended period of time, for example, a week, will reduce the time and costs required to retrieve these samples on a daily basis. In this manuscript, we addressed (1) the capacity of NIRS to predict the time post death of female *A. aegypti* mosquitoes left in a BG-Sentinel trap for a period of 0–7 days, (2) the ability of NIRS to detect ZIKV, CHIKV, and *Wolbachia* in *A. aegypti* mosquitoes left in a BG-Sentinel trap for 0–7 days, and (3) whether time post death of mosquitoes affected the capacity of NIRS to detect infections in mosquitoes.

In this study, we obtained 93.2, 97.0, and 90.3% accuracy when NIRS was used to predict ZIKV, CHIKV, and *Wolbachia* infection in female *A. aegypti* mosquitoes regardless of their time of death. More importantly, NIRS prediction of these infections 7 days post death was not significantly different from fresh mosquitoes. Surprisingly, NIRS showed higher accuracy than RT-qPCR for detecting ZIKV infection in dead mosquitoes. This could be likely due to the degradation of viral RNA at insectary temperature. We hypothesize that the rate of water loss in a mosquito in the presence of an arbovirus might be slower than viral RNA degradation. This allows NIRS to detect these viruses more efficiently than molecular-based approaches, such as the RT-qPCR^48,49^. NIRS has previously predicted the presence of ZIKV in heads and thoraces of female *A. aegypti* mosquitoes with 97% accuracy^37^, malaria parasites in *A. gambiae* with 95% accuracy^50^, and the presence of *w*Mel and *w*MelPop in female *A. aegypti* mosquitoes with 93% and 96% accuracy, respectively^38^.Previous studies used either fresh^37,50^ or RNAlater-preserved mosquitoes^38^ that were reared, infected, and maintained in a temperature-controlled environment and killed with chemicals. Herein, we simulated a more natural setting whereby *A. aegypti* females were killed by shaking the trap, and then left in a BG-Sentinel trap for a period of 0–7 days following their death.

Slightly higher accuracy was observed for CHIKV-infected *A. aegypti* than for ZIKV-infected mosquitoes (Table 1). This difference in accuracy could be attributed to specific viral changes in mosquitoes following an infection^44,51,52^, initial titers used to infect mosquitoes and the overall chemical composition of a mosquito following an infection. These factors ultimately affect the diagnostic signature and the accuracy. We noted no linear changes on the predicted accuracy or sensitivity for all infections over the 7-day sampling period. For instance, the highest accuracy for ZIKV, CHIKV, and *Wolbachia* was observed 2, 6, and 7 days post death, respectively. The authors speculate that the rate of water loss following the death of a mosquito could be relative to the type of infection carried by that mosquito because at 7 days post death, all infections were predicted with a relatively higher accuracy compared to fresh mosquitoes possibly indicating the peak of water loss regardless of the infection type. Nonetheless, the >90% accuracy of NIRS for detecting ZIKV or CHIKV in trapped *A. aegypti* several days post death was impressive, and indicates the potential future application of the tool for this purpose.

The release of *Wolbachia*-infected *A. aegypti* has the potential to become a key component of vector control in the future. In the past decade, *Wolbachia* has been released in small isolated sites and in big cities. Rapid assays to evaluate its effect on a large scale is crucial for assessing its invasion rate^27,28,53^. Herein, we have demonstrated the ability of NIRS in detecting *Wolbachia* in female *A. aegypti* up to 7 days post death. Although >85% accuracy was achieved over the 7-day sampling period, the accuracy of NIRS in detecting *Wolbachia* was slightly lower than that for CHIKV- and ZIKV-infected *A. aegypti*. We hypothesize this could related to the differences between immunological responses against a virus and a bacterium by a mosquito, as well as the distribution of the virus and the bacteria inside the mosquito. The *w*Mel strain of the endosymbiont *Wolbachia* is present in higher densities in specific tissues, notably in the ovaries^54^. Spectra of infected and uninfected mosquitoes were collected from the heads and thoraces where the density of *Wolbachia* is still significant, but in lower densities than ovaries to mitigate the effects of abdominal contents on the spectra, which could have had an effect on the accuracy of *Wolbachia*-infected mosquitoes. Future work should investigate whether scanning other parts of the mosquito improves the current accuracy, and whether *Wolbachia* can also be detected in male mosquitoes several days post death of a mosquito.

Monitoring arboviruses circulation in trapped mosquitoes could significantly enhance the knowledge regarding silent transmission of DENV, ZIKV, and CHIKV in endemic settings^45,55–57^. We define silent transmission as infections that result in either asymptomatic cases or mild symptoms that are undetected by routine surveillance systems^58^. Silent infections hold a significant role in arbovirus transmission because these infections could account for >80% of DENV transmission^58^. Such findings could trigger the deployment of a trap-based surveillance system, whereby rapid detection of pathogens in trapped mosquitoes could inform health managers to conduct cross-sectional serosurveys on nearby households. By doing so, integrated vector management practices, such as social mobilization, empowerment of communities, intensification of vector control, and an assertive case management approaches could locally impact disease transmission.

Mosquitoes used in this experiment were killed by shaking to mimic natural death, i.e., without chemicals. The mosquitoes were added to the catching bag of a BG-Sentinel trap, a harsh microenvironment produced by the constantly blowing fan intended to retain trapped mosquitoes. NIRS predicted the time post death based on the reduction of moisture/water content over time. This can be observed in the spectra shown in Fig. 2, where fresh samples are characterized by broad overtones of water related peaks ~1450 and 1950 nm, and whereby these peaks start to diminish 7 days post death.

The results presented herein are a first step toward the development of a robust arboviruses surveillance system that could be used to predict infections in mosquitoes at different time points. Arboviruses detection under field settings would require additional experiments to account for fluctuating environmental conditions in the field, while mosquitoes are trapped. Furthermore, we acknowledge the fact that a single cohort of mosquitoes was used to develop predictive models for CHIKV. Additional experiments assessing this effect would be essential prior to utilization of NIRS within a field setting. It is expected that current models for ZIKV, CHIKV, and *Wolbachia* would require to be modified by including field samples in the training model to capture confounding factors, such as diet, microbiome, fluctuating temperature, and humidity prior to field application^59^. Therefore, a direct extrapolation of our results to a field setting must be avoided at this point. Nonetheless, these results are encouraging and will accelerate progress toward developing a rapid and high-throughput arbovirus surveillance system.

## Methods

### Mosquitoes

Mosquitoes were collected from two sites in Rio de Janeiro, Brazil. To represent the native field population, i.e., those with and without *Wolbachia*. *Wolbachia*-free mosquitoes were collected at Urca neighborhood (22° 56′ 43″ S; 43° 09′ 42″ W), whereas those infected with *Wolbachia* were collected in Tubiacanga (22° 47′ 06″ S; 43° 13′ 32″ W), an isolated village in Rio where *Wolbachia* was first deployed. Infection rates in Tubiacanga are close to 100% (ref. ^27^). To capture the local genetic diversity for both sites, collection was done using 60 ovitraps homogeneously distributed over the area^60^. Eggs were hatched and larvae reared at the insectarium of the Laboratório de Mosquitos Transmissores de Hematozoários, Fiocruz. Larvae were fed with TetraMin fish flakes (Tetra GmbH, Melle, Germany) until pupation. Pupae were transferred to cages measuring 40 cm^3^ and emerging adults received 10% sugar solution ad libitum. Adults were maintained in an insectary under 27 ± 2° C, 70 ± 5% relative humidity and a 12:12 h light:dark cycling period. For the experiment described below, four groups of mosquitoes were used: *Wolbachia*-infected, CHIKV-infected, ZIKV-infected, and wild-type (uninfected) *A. aegypti*.

### Experimental blood feeding and infection

F1 female *A. aegypti* that were 5–6 days old were orally fed with 1 ml of ZIKV-infected or CHIKV-infected C6/36 cells supernatant mixed with 2 ml of human blood. We used the currently circulating strain of the Brazilian ZIKV [BRPE243/2015 (BRPE)]^61^, which was isolated from a ZIKV-infected patient in late 2015 and maintained in cell culture. The East–Central–South African CHIKV genotype deposited in the GenBank under accession nos. KP164567–KP164572, which was isolated from a patient in 2014 (ref. ^62^) was used. Viral titers were quantified via plaque forming assay for both ZIKV and CHIKV prior to infection.

The oral infection procedures were performed through a membrane feeding system (Hemotek, Great Harwood, UK) adapted with a pig-gut covering, which gives access to the human blood. The ZIKV viral titer used for mosquitoes was 1.9 × 10^6^ PFU (plaque formation units)/ml and the CHIKV viral titer was 6.3 × 10^5^ PFU/ml. Uninfected and *Wolbachia*-infected mosquitoes were fed similarly with uninfected blood and C6/36 cell culture. At least 40 mosquitoes were used for each experiment type. Mosquitoes were incubated for 7 days to allow the virus to replicate within mosquito body^51,52^ and to allow oviposition to take place. Results presented herein were obtained from three independent experiments for *Wolbachia*, three for ZIKV, and from a single experimental infection for CHIKV. Uninfected mosquitoes were used in every single assay.

### Sample preparation and collection of NIRS spectra

Following a 7-day incubation period, mosquitoes were killed by repeated shaking to avoid use of chemicals that could influence spectra characteristics. Mosquitoes were shaken for 5 min, with a 5 min pause to observe recovery, followed by additional 5 min of shaking if needed. Mosquitoes were individually transferred to a grid with 20, 1.5 ml open ended plastic tubes covered with nylon mesh on both ends. The mesh allowed air circulation and simulated the air fan that operates continuously in a BG-Sentinel trap. Mosquitoes in the grid were placed into the catch bag of the BG-Sentinel trap for 7 days. The BG-Sentinel mosquito traps remained side-by-side in the insectary during the experiment, with standard conditions (temperature: 27 ± 2° C; relative humidity: 70 ± 5%; 12:12 h light:dark cycling period).

### Spectra collection

Once a day for the 7-day period, mosquitoes remained in the trap, *A. aegypti* females were removed from the plastic grid for scanning with NIRS. By doing so, we were able to determine the limit of detection of NIRS for ZIKV, CHIKV, or *Wolbachia* infection in individually trapped *A. aegypti* mosquitoes. The insects were arranged sideways on a Spectralon diffuse reflection stage, and their heads and thoraces were scanned with a Labspec 4i NIRS spectrometer (Malvern Panalytical, Longmont, CO) using an external 3.2 mm diameter fiber optic probe and a 18, 6 W light source (Model 135325 Rev B, ASD Inc.) according to previously published protocols^31^. Spectra collection was on average 3–5 s per sample.

### Confirmation of ZIKV, CHIKV, and *Wolbachia* in mosquitoes

Infected mosquitoes were screened by qPCR to assess their infection status. A subset of 59, 157 CHIKV-infected and ZIKV-infected mosquitoes were assessed using RT-qPCR on legs before they were placed into the BG-Sentinel. A second RT-qPCR was conducted on 97 ZIKV-infected mosquitoes following the 7-day trapping period. Viral RNA was extracted with a QIAamp Mini Viral RNA Kit (Qiagen). Detection and quantification of viral RNA in legs from each individual was performed by RT-qPCR with the SuperScript III Platinum Single-Step qRT-PCR Kit (Invitrogen), using the QuantStudio 6 Flex Real-Time PCR System (Applied Biosystems) according to published protocols with known primers and amplification conditions^63,64^. Viral copy numbers were calculated by absolute quantitation for each run, using a curve pattern in a six-point dilution series (10^1^–10^6^ copies) of in vitro ZIKV and CHIKV RNA transcripts^61^. The legs of *Wolbachia*-infected mosquitoes were not screened for *Wolbachia* since *w*Mel strain is known to be found in higher densities in *A. aegypti* ovaries and salivary glands compared to their legs^20^. A subset of 43 mosquito bodies were screened for *Wolbachia* by RT-qPCR after the 7 days in the BG-Sentinel trap. DNA extraction from *A. aegypti* body involved the addition of 50 μl buffer (10 mM Tris, 1 mM EDTA, 50 mM NaCl, pH 8.2) and 2 μl proteinase K followed by maceration for 30 s. Samples were incubated at 56° C and then at 98° C for 10 min each. A mixture containing the rps primers for mosquitoes and wsp for *Wolbachia* was used in the PCR mixture. Each agarose gel electrophoresis reaction contained a *Wolbachia* positive control (mosquitoes from a lab colony whose infection has been confirmed by PCR), a PCR-confirmed negative control, and a blank sample (distilled water). Extraction and PCR protocols were performed following published materials^20,27,29^. The PCR data was used as the gold standard, i.e., a mosquito that received an infected blood meal with a negative PCR result, was considered negative.

### Data analysis

Spectral data was analyzed in R version 3.6.2 (Dark and Stormy Night)^65^ using only samples confirmed positive by PCR. Partial least squares (PLS) regression was performed using the package “pls” and summary statistics were generated using the “caret” package. Data were initially encoded as reflectance and was converted to absorbance using Eq. (1).

1 $$A = \log \frac{1}{R}$$

Equation (1): conversion from reflectance to absorbance. *A* is the absorbance and *R* is the reflectance.

### Death grading

PLS regression was employed to predict the number of days post death for each sample (0–7 days). A second PLS regression model was developed to predict if samples fit into four categories: (a) freshly killed (day 0), (b) 1 day, (c) 2–4 days, or (d) >4 days post death. These groups were denoted as 0, 1, 2, and 3, respectively. Data were split into training (75%) and testing (25%) groups. The split was performed on a mosquito by mosquito basis, as such data from each mosquito for all days were assigned to either the training or testing set as a group.

The optimal number of factors used was identified computationally by optimizing the accuracy within the training dataset. *K*-fold cross validation (*k* = 5, reps = 10) was used to simultaneously train the optimal factor level and parameter weights. The model was optimized by minimizing the PLS root mean squared error (RMSE) between actual days post death and predicted days post death. To facilitate interpretation of results, the MAE instead of RMSE is reported for both the training and testing cohorts.

### Infectivity prediction

Partial least squares discriminative analysis (PLSDA) was employed for binary classification analyses, including infectivity prediction for ZIKV-infected vs. uninfected, *Wolbachia*-infected vs. uninfected, and CHIKV-infected vs. uninfected. PLSDA was also employed to develop a multi-class classification model simultaneously for ZIKV, *Wolbachia*, CHIKV, and uninfected samples. Data were analyzed individually day by day (i.e., one model for samples on day 0, another for day 1, etc.) and balanced for infectivity status within each cohort independently involving equal number of infected and uninfected samples per cohort. All samples not used to train the model were used to test the model. Infectivity was encoded using one-hot encoding (1 for infected, 0 for uninfected). Optimal factor level identification and parameter weight tuning was performed using the same method, as described for death grading.

One final model was generated to simultaneously differentiate between all conditions present in the study. The process for generating this model is similar to that used to generate the models for differentiating between any one disease and uninfected mosquitoes, however, instead of using one-hot encoding to specify disease as a binary outcome measure, the outcome variable was a multilevel categorical variable (ZIKV, CHIKV, *Wolbachia*, and uninfected). Accuracy instead of RMSE was used to optimize these models.

Accuracy, sensitivity, and specificity for the training and testing sets were reported where prediction scores >0.5 are considered infected and prediction scores <0.5 are considered uninfected.

### Monte Carlo simulation

To ensure the robustness of the models, Monte Carlo simulations were employed, and the procedure described above was repeated 50 times, randomly assigning mosquitoes to the training and testing groups differently in each repetition. Results reported describe aggregated results from all runs of the Monte Carlo simulation.

### Statistics and reproducibility

Statistical analysis was conducted on R (version 3.6.2—Dark and Stormy Night), using the packages “pls” and “caret” (as described above). The sample sizes for each infection treatment is described above and was based on previous experiments conducted at corresponding author’s laboratory to investigate the presence of ZIKV in freshly infected *A. aegypti*^37^. Methodology and biological materials are disclosed as much as possible, but if required, further information can be obtained by contacting from the corresponding author. The replicates of infected and control mosquitoes used in a given experimental infection were hatched at different days and raised under the same controlled conditions.

### Reporting summary

Further information on research design is available in the Nature Research Reporting Summary linked to this article.

## Supplementary information

Description of Supplementary Files

Supplementary Data 1

Supplementary Data 2

Supplementary Data 3

Supplementary Data 4

Supplementary Data 5

Reporting Summary

## Data Availability

The Source data generated during and/or analyzed during the current study are available as Supplementary Data 1–5. All other data, if any, will be available upon reasonalble request.
