# Supplementary material for: High throughput estimates of Wolbachia, Zika and chikungunya infection in Aedes aegypti by near-infrared spectroscopy to improve arbovirus surveillance
Source: Commun Biol. 2021 Jan 15;4:67. doi: 10.1038/s42003-020-01601-0 (PMC7810739; doi:10.1038/s42003-020-01601-0)
Supplement: Supplementary file 1 — Description of Supplementary Files [file 42003_2020_1601_MOESM1_ESM.pdf]

Description of Additional Supplementary Files

File name: Supplementary Data 1
Description: Source data for Figure1_training data

File name: Supplementary Data 2
Description: Source data for Figure1_testing data

File name: Supplementary Data 3
Description: Source data for Figure3_training data

File name: Supplementary Data 4
Description: Source data for Figure3_testing data

File name: Supplementary Data 5
Description: Raw data used to generate Figure 4.
