# Supplementary material for: High throughput estimates of Wolbachia, Zika and chikungunya infection in Aedes aegypti by near-infrared spectroscopy to improve arbovirus surveillance
Source: Commun Biol. 2021 Jan 15;4:67. doi: 10.1038/s42003-020-01601-0 (PMC7810739; doi:10.1038/s42003-020-01601-0)
Supplement: Supplementary file 7 — Reporting Summary [file 42003_2020_1601_MOESM7_ESM.pdf]

nature research

Corresponding author(s): Rafael Maciel-de-Freitas

Last updated by author(s): 16/10/20

Reporting Summary

Nature Research wishes to improve the reproducibility of the work that we publish. This form provides structure for consistency and transparency in reporting. For further information on Nature Research policies, see our Editorial Policies and the Editorial Policy Checklist.

Statistics

For all statistical analyses, confirm that the following items are present in the figure legend, table legend, main text, or Methods section.

n/a	Confirmed	
☐	☒	The exact sample size (*n*) for each experimental group/condition, given as a discrete number and unit of measurement
☐	☒	A statement on whether measurements were taken from distinct samples or whether the same sample was measured repeatedly
☐	☒	The statistical test(s) used AND whether they are one- or two-sided<br>*Only common tests should be described solely by name; describe more complex techniques in the Methods section.*
☐	☒	A description of all covariates tested
☐	☒	A description of any assumptions or corrections, such as tests of normality and adjustment for multiple comparisons
☐	☒	A full description of the statistical parameters including central tendency (e.g. means) or other basic estimates (e.g. regression coefficient) AND variation (e.g. standard deviation) or associated estimates of uncertainty (e.g. confidence intervals)
☐	☒	For null hypothesis testing, the test statistic (e.g. *F*, *t*, *r*) with confidence intervals, effect sizes, degrees of freedom and *P* value noted<br>*Give P values as exact values whenever suitable.*
☒	☐	For Bayesian analysis, information on the choice of priors and Markov chain Monte Carlo settings
☒	☐	For hierarchical and complex designs, identification of the appropriate level for tests and full reporting of outcomes
☒	☐	Estimates of effect sizes (e.g. Cohen's *d*, Pearson's *r*), indicating how they were calculated

Our web collection on statistics for biologists contains articles on many of the points above.

Software and code

Policy information about availability of computer code

Data collection

Data was collected using a Labspec 4i (Malvern Panalytical, Boulder, Colorado, USA) using a fibre optic external probe with six illumination fibres using an ASD software (Malvern Panalytical) and a labptop. Prior to spectral collection, the instrument was calibrated using a spectrralon white disc (Malvern Panalytical). All spectra were collected in reflectance mode within the 350-2500nm wavelength region.

Data analysis

Preprocessing:
Data were analysed using R version 3.6.1. Spectral data were converted from ASD format to SPC format using the prospectr package (v. 0.1.3) in R. Data were converted from reflectance to absorbance by applying the a=1/log(R) formula. Infections (confirmed by PCR) were converted to binary variables using one hot encoding. Days post death were considered as a continuous variable and recorded using whole numbers.

Machine learning:
Machine learning was performed in R using the following packages:
pls (v. 2.7.1)
caret (v. 6.0-84)
dplyr (v. 0.8.3)
imputeTS (no listed version number)
Figures 1, 3 and 4 were generated in R while figure 2 was generated using GraphPad Prism (v. 7)

Data were separated into training (75%) and testing (25%) sets. Monte carlo simulations (n=50) were performed to ensure the robustness of the findings. Data was stratified according to days post death and confirmed infection. Equal numbers of samples were used from these stratified groups to train the models. For each biological organism, all technical replicates were randomised as a block. Parameter tuning was performed on the training data only. Accuracy was defined by comparing predicted infectivity (>= 0.5) to PCR results.

Code used to generate the results presented in this manuscript is available upon request.

For manuscripts utilizing custom algorithms or software that are central to the research but not yet described in published literature, software must be made available to editors and reviewers. We strongly encourage code deposition in a community repository (e.g. GitHub). See the Nature Research guidelines for submitting code & software for further information.

Data

Policy information about availability of data

All manuscripts must include a data availability statement. This statement should provide the following information, where applicable:
- Accession codes, unique identifiers, or web links for publicly available datasets
- A list of figures that have associated raw data
- A description of any restrictions on data availability

All raw data is available for public access through figures supplied in the manuscript. Additional data is available through the corresponding author

Field-specific reporting

Please select the one below that is the best fit for your research. If you are not sure, read the appropriate sections before making your selection.

[x] Life sciences [ ] Behavioural & social sciences [ ] Ecological, evolutionary & environmental sciences

For a reference copy of the document with all sections, see nature.com/documents/nr-reporting-summary-flat.pdf

Life sciences study design

All studies must disclose on these points even when the disclosure is negative.

Sample size	Two replicates were performed for this experiment. Each replicate included approximately 60 mosquitoes with each infection. This number was chosen based on prior experience developing models for mosquito infections using NIRS. Chikungunya was included in the second experiment only, hence having approximately half the number of mosquitoes
Data exclusions	No data were excluded from these analyses
Replication	Two replication experiments were performed
Randomization	Randomization was not performed. Mosquitoes for each experiment were raised as a single colony. To control for influences of age, all mosquitoes included in each experiment were born on the same day. Environmental factors were controlled as each colony was maintained in laboratory conditions.
Blinding	As these mosquitoes were infected by the investigators and the local investigators on site needed to be aware of potential pathogens for safety reasons blinding was not performed.

Reporting for specific materials, systems and methods

We require information from authors about some types of materials, experimental systems and methods used in many studies. Here, indicate whether each material, system or method listed is relevant to your study. If you are not sure if a list item applies to your research, read the appropriate section before selecting a response.

Materials & experimental systems

n/a	Involved in the study	
[x]	[ ]	Antibodies
[x]	[ ]	Eukaryotic cell lines
[x]	[ ]	Palaeontology and archaeology
[ ]	[x]	Animals and other organisms
[x]	[ ]	Human research participants
[x]	[ ]	Clinical data
[ ]	[x]	Dual use research of concern

Methods

n/a	Involved in the study	
[x]	[ ]	ChIP-seq
[x]	[ ]	Flow cytometry
[x]	[ ]	MRI-based neuroimaging

Animals and other organisms

Policy information about studies involving animals; ARRIVE guidelines recommended for reporting animal research

Laboratory animals	First generation larvae of Aedes aegypti mosquitoes were reared in the laboratory and only 5-6 days old female mosquitoes were used in the study

Wild animals | The study did not involve wild animals

Field-collected samples | Aedes aegypti eggs were collected in the field using ovitraps and allowed to hatch in the lab at Laboratório de Mosquitos Transmissores de Hematozoários, Fiocruz. Larvae were fed with TetraMin fish flakes (Tetra Melle) until pupation. Pupae were transferred to cages and emerging adults received 10% sugar solution ad libitum. Adults were maintained under 27 °C, 70 % relative humidity and a 12:12 hours light:dark cycling period.

Ethics oversight | Fiocruz, Rio de Janeiro

Note that full information on the approval of the study protocol must also be provided in the manuscript.

Dual use research of concern

Policy information about dual use research of concern

Hazards

Could the accidental, deliberate or reckless misuse of agents or technologies generated in the work, or the application of information presented in the manuscript, pose a threat to:

No	Yes	
☒	☐	Public health
☒	☐	National security
☒	☐	Crops and/or livestock
☒	☐	Ecosystems
☒	☐	Any other significant area

Experiments of concern

Does the work involve any of these experiments of concern:

No	Yes	
☒	☐	Demonstrate how to render a vaccine ineffective
☒	☐	Confer resistance to therapeutically useful antibiotics or antiviral agents
☒	☐	Enhance the virulence of a pathogen or render a nonpathogen virulent
☒	☐	Increase transmissibility of a pathogen
☒	☐	Alter the host range of a pathogen
☒	☐	Enable evasion of diagnostic/detection modalities
☒	☐	Enable the weaponization of a biological agent or toxin
☒	☐	Any other potentially harmful combination of experiments and agents
